# Quality Without Compromise: A Propensity Score-Matched Analysis of Robotic Versus Laparoscopic Surgery for Locally Advanced Colorectal Cancer

**DOI:** 10.3390/cancers18101601

**Published:** 2026-05-14

**Authors:** Marcin Kubiak, Wojciech Górski, Radosław Mlak, Zuzanna Dąbrowska, Jolanta Sado, Kinga Bielarska, Szymon Bielecki, Karol Rawicz-Pruszyński, Katarzyna Sędłak

**Affiliations:** 1 Department of Surgical Oncology, Medical University of Lublin, Staszica 16 St., 20-081 Lublin, Polandkatarzyna.sedlak@umlub.edu.pl (K.S.); 2 Department of Laboratory Diagnostics, Medical University of Lublin, Chodźki 1 St., 20-093 Lublin, Poland

**Keywords:** colorectal cancer, robotic surgery, laparoscopic surgery, textbook outcome, propensity score matching, oncological outcome

## Abstract

Background: Minimally invasive surgery is the standard approach in colorectal cancer (CRC), yet the clinical value of robotic-assisted surgery (RAS) compared with laparoscopy remains under debate. This study aimed to compare surgical quality using textbook outcome (TO) and textbook oncological outcome (TOO) in patients undergoing minimally invasive resection for locally advanced CRC. Methods: A retrospective analysis of patients with locally advanced CRC (cT2-4N0-2M0) treated in a high-volume centre was performed. Patients undergoing laparoscopic or robotic surgery were included. Propensity score matching (PSM) was applied to balance baseline characteristics. TO was defined as an optimal perioperative course without complications, conversion, reintervention, prolonged length of stay, or mortality. TOO extended TO by including oncological parameters such as R0 resection and adequate lymph node yield. Results: A total of 123 patients were included (80 laparoscopic, 43 robotic), with 80 patients analyzed after PSM (40 per group). RAS was associated with significantly higher rates of intracorporeal (97% vs. 18.9%) and mechanical anastomoses (96.9% vs. 48.6%). No differences were observed in postoperative complications, reintervention rates, length of stay, or mortality. Although the comprehensive complication index was lower in the robotic group, this did not translate into improved TO or TOO rates. After matching, TO was achieved in 72.5% of RAS and 85.0% of laparoscopic cases (*p* = 0.1745), while TOO rates were also comparable between groups. No independent predictors of TO or TOO were identified in multivariable analysis. Conclusions: Robotic surgery for locally advanced CRC provides comparable perioperative safety and oncological quality to laparoscopy. The implementation of RAS in a high-volume centre does not compromise short-term outcomes, supporting its safe integration into clinical practice.

## Simple Summary

Colorectal cancer (CRC) is one of the most common cancers worldwide, and surgery remains the main treatment. Minimally invasive techniques, such as laparoscopic and robotic surgery, are widely used, but it is still unclear whether robotic surgery improves overall treatment quality. In this study, we compared these two approaches in patients with locally advanced CRC treated in a high-volume centre. We assessed both short-term recovery and the quality of cancer surgery using the textbook outcome and the textbook oncological outcome. Our results showed that robotic surgery is as safe and effective as laparoscopic surgery, even during the early stage of its introduction. These findings suggest that robotic surgery can be safely implemented in clinical practice without compromising patient outcomes.

## 1. Introduction

According to GLOBOCAN estimates, colorectal cancer (CRC) accounts for approximately 1.9 million new cases annually, ranking third worldwide in cancer incidence. With an estimated 904,000 deaths per year, CRC represents the second leading cause of cancer-related mortality globally [1]. While CRC is most commonly diagnosed in Australia, New Zealand and Western Europe, the highest mortality rates are observed in Eastern Europe [2]. It is estimated that by 2040, the number of new cases of colorectal cancer will rise to 3.2 million, and the number of deaths from the disease will reach 1.6 million. It is predicted that the majority of cases will occur in countries with a high or very high human development index (HDI) [2]. The results of randomized controlled trials (RCTs) and recent years of clinical experience have shown that laparoscopic surgery for CRC is both surgically safe and oncologically adequate [3,4]. It was only natural to attempt to draw similar conclusions regarding robotic-assisted surgery (RAS) [5,6]. The potential benefits of surgical RAS have been repeatedly demonstrated, such as precise work in a limited surgical field, potentially less blood loss and reduced surgical trauma. This is particularly evident in centres with extensive experience in minimally invasive surgery, where shorter length of stay (LOS) and lower minor complication rates have been demonstrated in patients who underwent RAS in association with the enhanced recovery after surgery (ERAS) protocol [7]. For rectal cancer (RC) surgery, the superiority of RAS over laparoscopic surgery in terms of oncological outcome has also been demonstrated, as shown in ROLARR and REAL studies [5,8]. Thanks to the continuous advancement of robotic surgery, with the introduction of new technologies such as Single Port robots, and the increasingly widespread use of artificial intelligence, these results are likely to become even more promising [9,10]. However, regardless of the surgical approach, international guidelines consistently define the primary goal of surgery for locally advanced CRC as complete resection with negative microscopic margins and adequate lymphadenectomy [11,12,13]. For over a decade, the concept of textbook outcome (TO) has been one of the basic and widely used measures of surgical treatment quality [14,15]. TO is typically defined by a composite of the following parameters: absence of intraoperative and postoperative complications, no perioperative mortality, no prolonged LOS, and no reintervention and was widely used in the case of CRC surgery [16,17]. Currently, CRC treatment is based on multimodal treatment [13,18]. Therefore, a textbook oncological outcome (TOO) has also been proposed in the context of most gastrointestinal tract cancer treatments [19,20,21]. In the case of CRC, the definition of TOO comprises all TO parameters and indicators directly influencing the oncological prognosis: a negative resection margin and, in the case of CC, the removal of at least 12 lymph nodes. Considering the above, the aim of our study was to compare TO and TOO in robotic and laparoscopic colorectal surgery among patients treated in a high-volume centre in Poland.

## 2. Methods

### 2.1. Study Area, Study Design, Data Source and Patient Selection

**Figure 1:**
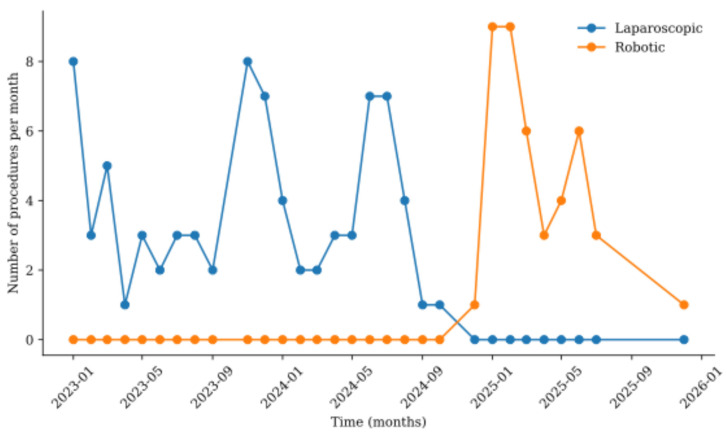
The monthly number of laparoscopic and robotic procedures over time.

This single-centre study was conducted at a university hospital in a city of 300,000 people in Eastern Poland. Approximately 150 CRC patients are treated at the unit each year, which qualifies it as a high-volume centre [22]. A total of 123 patients with histologically confirmed locally advanced CRC (cT2-4N0-2M0) who underwent minimally invasive surgical treatment with curative intent between 2023 and 2025 were included in the study. All patients who underwent laparoscopic surgery between 2023 and 2025 were enrolled in the study. During the study period, the surgical approach was determined mainly by institutional availability and implementation of the robotic programme. Laparoscopic surgery represented the standard minimally invasive approach before the introduction of robotic colorectal surgery. Robotic surgery was introduced in December 2024; thereafter, patients eligible for minimally invasive resection were preferentially considered for robotic surgery according to MDT decision-making, surgeon assessment, and robotic system availability. Thus, laparoscopic and robotic procedures were performed predominantly in sequential time periods rather than as two fully concurrent treatment options (Figure 1).

**Figure 2:**
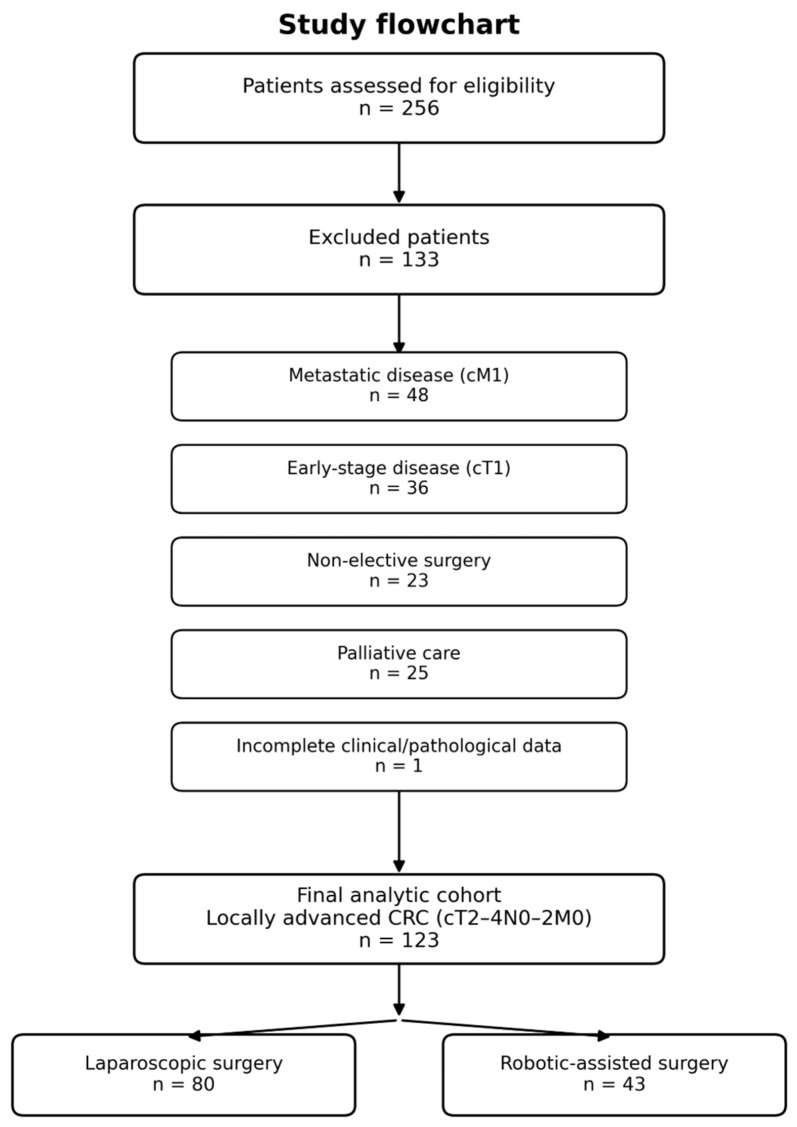
Flowchart of the study.

The exclusion criteria comprised: open surgery, non-elective surgery, disease at an early (cT1) or metastatic stage (cM1) at the time of diagnosis, patients receiving palliative care, or those with incomplete clinical or pathological data that would preclude analysis (Figure 2).

All patients were managed according to current international guidelines for CRC, including multidisciplinary team (MDT) decision-making and appropriate perioperative oncological treatment strategies. The study was conducted in accordance with the Strengthening the Reporting of Observational Studies in Epidemiology (STROBE) guidelines [23]. Approval was obtained from the appropriate Institutional Review Board (KE-0254/331/2018), and all procedures were performed in accordance with the Declaration of Helsinki [24].

### 2.2. Perioperative Management and Oncological Treatment

Patients were qualified for treatment based on clinical staging, including imaging modalities (computed tomography in CC patients, magnetic resonance imaging in RC patients). For patients with locally advanced RC, neoadjuvant treatment, including radiotherapy, chemoradiotherapy or total neoadjuvant treatment (TNT), was administered according to tumour stage and institutional protocols. Perioperative systemic therapy was applied selectively in accordance with current guidelines. Surgical treatment was performed following completion of neoadjuvant therapy when indicated, with appropriate intervals to allow for tumour response and patient recovery.

### 2.3. Surgical Procedures

All patients underwent minimally invasive resection. Surgical techniques adhered to established oncological principles, including complete mesocolic excision (CME) for CC and total mesorectal excision (TME) for RC, with high vascular ligation performed when appropriate. The primary surgical objective in all cases was en bloc tumour resection with negative macroscopic and microscopic margins and adequate lymphadenectomy. Conversion to open surgery was recorded and included as a variable in outcome assessment (Table 1).

### 2.4. Variables and Definitions

Clinicopathological data included patient demographics such as age and sex, tumour characteristics including location (colon vs. rectum), clinical and pathological TNM stage, and grading, as well as treatment-related variables such as surgical approach and use of neoadjuvant therapy. Age was included as a continuous variable, while sex, tumour location, clinical stage, and neoadjuvant therapy were treated as categorical variables. Postoperative complications were assessed using standardized criteria, and their severity was graded according to the Clavien–Dindo classification [25]. Additionally, the comprehensive complication index (CCI) was calculated to provide an overall measure of postoperative morbidity [26].

### 2.5. Outcomes

The primary endpoints of this study were TO and TOO, selected as comprehensive composite measures reflecting both perioperative safety and oncological quality. These endpoints were chosen to directly correspond with the study objective, which was to compare the overall quality of surgical treatment between robotic-assisted and laparoscopic approaches.

TO was defined as an optimal perioperative course and included all of the following criteria: absence of intraoperative and postoperative complications, no conversion to open surgery, no need for reintervention, no perioperative mortality, and LOS shorter than 14 days. This composite endpoint was used to provide a standardized and clinically meaningful assessment of short-term surgical quality.

TOO was defined as an extension of TO, incorporating additional parameters reflecting oncological adequacy of the procedure. In addition to fulfilling all TO criteria, TOO required a microscopically margin-negative (R0) resection and, in cases of colon cancer, retrieval of at least 12 lymph nodes, in accordance with established oncological standards.

The follow-up period after surgery was 90 days, and the absence of postoperative complications was assessed within 30 days of the procedure.

Both TO and TOO were analyzed as binary outcomes (achieved vs. not achieved) and were used consistently across all statistical analyses, including comparisons between surgical approaches and regression models assessing factors associated with their attainment. This approach allowed for an integrated evaluation of surgical performance, combining perioperative and oncological dimensions into unified outcome measures.

### 2.6. Propensity Score Matching (PSM)

To minimize selection bias and account for baseline differences between the laparoscopic and robotic groups, PSM was performed. Matching was based on clinically relevant variables, including age, sex, tumour location, clinical stage, and use of neoadjuvant therapy. Patients were matched using a nearest-neighbour method in a 1:1 ratio without replacement. The balance between matched groups was assessed using standardized mean differences.

Following PSM, the cohort was analyzed as a balanced sample, and standard logistic regression models were applied rather than conditional models, as the matching was performed to achieve covariate balance rather than to establish fixed matched pairs.

The analysis was interpreted as exploratory. Given the limited number of endpoint failures in the matched cohort, post-matching regression modelling was kept limited to avoid unstable estimates and overfitting. Therefore, the matched cohort was primarily used to compare the distribution of outcomes and endpoint components between the surgical approaches.

No formal a priori sample size calculation was performed because of the retrospective design of the study. The sample size was determined by the number of consecutive patients who met the predefined eligibility criteria during the study period. Therefore, the analysis should be interpreted as exploratory.

### 2.7. Statistical Analysis

Categorical variables were presented as absolute numbers and percentages, whereas continuous variables were expressed as mean values with standard deviation or as medians with interquartile ranges, depending on data distribution. Comparisons between groups were performed using the chi-square or Fisher’s exact test for categorical variables and Student’s *t*-test or Mann–Whitney U test for continuous variables, as appropriate. Univariable and multivariable analyses were conducted to identify factors associated with achieving TO and TOO, using logistic regression models to estimate odds ratios with corresponding 95% confidence intervals. All statistical tests were two-sided, and a *p*-value below 0.05 was considered statistically significant. Statistical analyses were performed using appropriate statistical software.

## 3. Results

A total of 123 patients who underwent minimally invasive surgery for locally advanced CRC were included in the final analytic cohort, including 80 patients treated laparoscopically and 43 undergoing RAS. The median age at diagnosis was comparable between the laparoscopic and robotic groups (63.5 vs. 67 years, respectively) (Table 1).

Before matching, differences between the groups reflected the evolving adoption of RAS and potential selection bias. After PSM, 80 patients were included in the final analysis, with 40 patients in each arm. The matched groups were well balanced in terms of baseline clinicopathological characteristics, including age, sex, tumour location (CC vs. RC), clinical TNM stage, and use of neoadjuvant therapy, confirming adequate adjustment for tumour burden and patient-related factors.

RAS was associated with a significantly higher rate of intracorporeal anastomosis compared with laparoscopy (97% vs. 18.9%, *p* < 0.0001). Similarly, mechanical anastomosis was more frequently performed in the robotic group (96.9% vs. 48.6%, *p* < 0.0001) (Table 2 and Table 3).

The incidence of postoperative complications, reinterventions, length of stay (LOS), and perioperative mortality did not differ significantly between RAS and laparoscopic surgery. Although the median comprehensive complication index (CCI) was lower in the robotic group (0 vs. 8.7, *p* < 0.001), this difference did not translate into higher rates of TO or TOO (Table 4). In univariable analysis, a higher CCI was observed in patients who did not achieve TO or TOO compared with those who did (20.9 vs. 0; *p* < 0.0001), whereas in multivariable analysis, no independent predictors of TO were identified.

After PSM, the rate of TO was comparable between the laparoscopic and robotic groups (85.0% vs. 72.5%, *p* = 0.1745) (Table 5). In univariable analysis, no consistent clinicopathological factors were associated with achieving TO, and these findings were confirmed in multivariable analysis, where none of the analyzed variables independently affected the likelihood of achieving this endpoint.

Similarly, no significant differences were observed in TOO between the two approaches (Table 4 and Table 5). In univariable analysis, rectal tumour location was associated with a higher likelihood of achieving TOO compared with colon tumours (75% vs. 28.6%; *p* = 0.0247), whereas more advanced clinical stage (cT3–4) was associated with a lower probability of achieving TOO compared with cT1–2 tumours (100% vs. 42.3%; *p* = 0.0052). However, in multivariable analysis, none of these variables remained statistically significant, indicating the lack of independent predictors of TOO.

## 4. Discussion

Although minimally invasive approaches are standard in CRC surgery, demonstrating a potential advantage of RAS over laparoscopy remains challenging, particularly in countries such as Poland, where the relatively late adoption of RAS may both limit availability and influence outcome comparisons. Similarly, questions remain as to whether it is possible to safely introduce RAS without oncological consequences for the patient, particularly when there are no national registries to oversee and monitor the process of integrating RAS into everyday practice [27]. In the present PSM analysis, our main finding was that implementation of RAS was as safe as laparoscopy regarding postoperative morbidity, while maintaining similar oncological quality. The rate of TO was 72.5% after RAS vs. 85.0% after laparoscopy, whereas TOO was achieved in 55.9% and 79.4% of patients, respectively. At the same time, the two approaches yielded similar oncological surrogates, including lymph node retrieval and the rate of radical resection, while rates of reintervention, ICU stay, anastomotic leak, and perioperative mortality remained low in both groups. In addition, RAS was associated with a different technical profile, with a markedly higher utilization of intracorporeal anastomosis (97.0% vs. 18.9%) and mechanical anastomosis (96.9% vs. 48.6%). These data suggest that, in high-volume centres, RAS introduction does not compromise short-term safety or oncological adequacy relative to laparoscopy.

Our findings are consistent with the best available randomized evidence in RC, which overall supports at least equivalence between robotic and laparoscopic surgery for key perioperative and oncological endpoints while suggesting potential technical advantages of RAS [28,29]. In the ROLARR trial (Jayne et al., 2017 [3]), which randomized 471 patients with RC, conversion to open surgery occurred in 8.1% of patients undergoing RAS compared with 12.2% after laparoscopy. Although this difference did not reach significance, the absolute reduction consistently favoured RAS and may reflect improved technical feasibility during pelvic dissection. Likewise, the rate of positive circumferential resection margins remained lower in the robotic group (5.1% vs. 6.3%; *p* = 0.77), indicating at least comparable oncological adequacy. Notably, exploratory analyses suggested that the benefit of RAS may become more evident in technically demanding scenarios, particularly in male or obese patients, and patients with low RC, where the restricted pelvic workspace may limit laparoscopic maneuverability. At the same time, no gender differences were observed among patients in our cohort. In this context, the improved visibility and greater precision of instrument movements that are characteristic of RAS can facilitate precise TME procedures whilst maintaining oncological standards [5].

At the same time, more recent randomized controlled trial (RCT) indicates that RAS may confer advantages in selected RC settings. In the REAL trial, 1171 patients with middle and low RC were analyzed, and the 3-year locoregional recurrence rate was 1.6% after RAS vs. 4.0% after laparoscopy, while 3-year disease-free survival (DFS) was 87.2% vs. 83.4%, respectively [8]. Although long-term oncological outcomes, such as DFS or recurrence-free survival (RFS), were beyond the scope of this study, the comparable R0 resection rates and lymph node yields observed with RAS are in line with the oncological quality signal reported in the REAL study. This interpretation is further supported by recent meta-analyses restricted to RCTs in RC. Tang et al. pooled 7 RCTs including 507 robotic and 516 laparoscopic rectal resections and found no differences in overall 30-day postoperative complications, severe complications, anastomotic leakage, conversion, reoperation or perioperative mortality [30]. Likewise, the updated RCT-only meta-analysis by Zou et al., which included 11 RCTs and 3107 patients (1552 robotic and 1555 laparoscopic procedures), reported no significant differences between robotic and laparoscopic surgery in several perioperative outcomes. Specifically, there was no difference in overall postoperative complications, short-term postoperative complications, estimated blood loss, LOS, intraoperative complications, perioperative mortality or readmission. Nonetheless, RAS was associated with lower conversion rates, lower reintervention rates, higher lymph node yield and lower rate of positive circumferential resection margins, although RAS was associated with longer operative time. This may subsequently lead to better long-term and oncological outcomes.

Comparable findings have also been reported in studies focusing on CC. In a large institutional cohort analysis, Yuval et al. (2023) demonstrated that robotic colectomy achieved oncological outcomes comparable to laparoscopic surgery, including similar lymph node yield and rates of radical resection, while differences between the approaches were mainly limited to perioperative outcomes [4]. Similarly, in a European study evaluating CC patients with pT4 tumours, de’Angelis et al. (2024) [31] reported comparable oncological adequacy between robotic and laparoscopic right colectomy, with R0 resection rates of 92.3% and 96.2%, respectively, and retrieval of at least 12 lymph nodes in 97.4% vs. 96.2% of cases. At the same time, RAS was associated with lower conversion rates (5.1% vs. 20.5%), reduced blood loss, fewer postoperative complications (17.9% vs. 41.0%), and shorter LOS (6.4 vs. 9.5 days) [31]. A similar conclusion was reported in the recent meta-analysis by Chaouch et al. (2025) [6], which included 733 patients undergoing right colectomy with CME. In this analysis, robotic and laparoscopic surgery demonstrated comparable perioperative outcomes, including postoperative morbidity, estimated blood loss, LOS and lymph node yield. Moreover, a lower conversion rate has been demonstrated in RAS (2.7% vs. 6.5%; *p* = 0.04) [6].

The present study should also be viewed in the context of the emerging literature on TO in colorectal surgery. In the Dutch population-based study of 20,521 RC patients, Warps et al. reported a TO rate of 56.3%, and postoperative complications were identified as the main reason for failure to achieve TO. Notably, laparoscopic surgery was positively associated with TO in multilevel analysis [32]. In CC surgery, Manatakis et al. reported a TO rate of 60.2%, with the lowest-performing individual components being “no major complications” (69.5%) and “no prolonged LOS” (75%). For comparison, in our study, the TO rate was 72.5% for RAS and 85% for laparoscopic surgery. Importantly, 5-year overall survival (OS) was 81% in the TO group vs. 59% in the non-TO group, and 5-year cancer-specific survival was 86% vs. 65% [33].

Importantly, our findings should also be interpreted in the context of the conceptual framework outlined in the introduction, where composite quality metrics such as TO and TOO were presented as integrative measures of surgical performance in CRC. While the existing literature has predominantly focused on isolated perioperative or oncological endpoints, the clinical reality of CRC management increasingly requires multidimensional assessment strategies that capture both domains simultaneously. In this regard, our study provides additional evidence that RAS can achieve comparable overall treatment quality when evaluated using these composite metrics, even during the early phase of programme implementation. The consistency of TO and TOOs between approaches observed in our cohort supports the growing paradigm shift towards comprehensive, patient-centred quality assessment and highlights the relevance of composite endpoints as robust tools for benchmarking surgical performance in contemporary CRC practice.

Several limitations of the present study should be acknowledged. First, the retrospective design introduces the possibility of residual selection bias despite the use of PSM. Second, the sample size after matching was relatively modest, making composite endpoints such as TO and TOO sensitive to variation in individual components. Third, the cohort included both CC and RC, introducing heterogeneity related to anatomy, neoadjuvant therapy, and the interpretation of lymph node yield. Fourth, the follow-up duration was insufficient to evaluate long-term oncological outcomes, including DFS or OS. Finally, the definition and interpretation of TOO in CRC remain incompletely standardized, particularly in RC after neoadjuvant therapy, where lymph node yield may be biologically altered, and margin status may carry greater prognostic significance. The sample size after PSM was modest, with 40 matched pairs available for the primary TO analysis. Consequently, the study was underpowered to detect small-to-moderate differences between laparoscopic and robotic surgery. For example, the observed absolute difference in TO rates after matching was 12.5 percentage points, whereas a substantially larger effect would be required to achieve adequate statistical power in a cohort of this size. Therefore, the lack of statistically significant differences should be interpreted as the absence of a detected difference rather than evidence of formal equivalence or non-inferiority. Given the modest number of matched pairs and endpoint failures, post-matching regression estimates should be interpreted cautiously. The present study was not powered to provide formal evidence of equivalence or non-inferiority between robotic and laparoscopic surgery.”

Despite these limitations, the study provides clinically relevant insights. It reflects contemporary practice from a high-volume centre during the early, structured implementation of robotic colorectal programmes in a healthcare system currently experiencing a rapid expansion of RAS. Notably, in Poland, the number of hospitals equipped with robotic systems nearly doubled between 2022 and 2023, reaching approximately 60 centres, highlighting the dynamic adoption of this technology [27]. Importantly, to the best of our knowledge, this represents one of the first analyses from Poland evaluating the introduction of robotic colorectal surgery in a real-world setting. The study comprehensively assesses both perioperative and oncological quality using composite endpoints. Most importantly, it demonstrates that robotic colorectal surgery can be implemented without compromising short-term safety or oncological adequacy when compared with laparoscopy, even during the initial phase of adoption.

## 5. Conclusions

RAS for locally advanced CRC was as safe as laparoscopy in terms of postoperative morbidity and overall complication burden, while maintaining similar oncological quality as reflected by lymph node retrieval and radical resection rates.

## Figures and Tables

**Table 1:** Comparison of demographic and clinical variables depending on the surgical technique (before/after PSM).

Variable	TO Before PSM [N = 123]		*p*	TO After PSM [N = 80]		*p*	TOO Before PSM [N = 109]		*p*	TOO After PSM [N = 68]		*p*
	Laparoscopy [N = 80] N (%) or Median [IQR] (Min.–Max.)	Robot [N = 43] N (%) or Median [IQR] (Min.–Max.)		Laparoscopy [N = 40] N (%) or Median [IQR] (Min.–Max.)	Robot [N = 40] N (%) or Median [IQR] (Min.–Max.)		Laparoscopy [N = 72] N (%) or Median [IQR] (Min.–Max.)	Robot [N = 37] N (%) or Median [IQR] (Min.–Max.)		Laparoscopy [N = 34] N (%) or Median [IQR] (Min.–Max.)	Robot [N = 34] N (%) or Median [IQR] (Min.–Max.)	
**Sex**			0.9025			1.0000			0.4183			1.0000
Female	40 (50%)	22 (51.2%)		21 (52.5%)	21 (52.5%)		33 (45.8%)	20 (54.1%)		19 (55.9%)	19 (55.9%)	
Male	40 (50%)	21 (48.8%)		19 (47.5%)	19 (47.5%)		39 (54.2%)	17 (45.9%)		15 (44.1%)	15 (44.1%)	
**Age (median)**	63.5 [56–70.5] (28–81)	67 [59.3–75] (42–83)	0.0402 *	65 (56.5–70] (46–80)	67 [59.5–74.5] (42–80)	0.1497	64 [57–71] (28–81)	69 [60–75] (42–83)	0.0793	64 [59–60] (47–80)	67 [60–74] (42–80)	0.1910
**Age**			0.1526			0.4975			0.1079			0.3301
<65 years	42 (52.5%)	16 (37.2%)		19 (47.5%)	15 (37.5%)		33 (45.8%)	23 (62.2%)		18 (52.9%)	13 (38.2%)	
≥65 years	38 (47.5%)	27 (62.8%)		21 (52.5%)	25 (62.5%)		39 (54.2%)	14 (37.8%)		16 (47.1%)	21 (61.8%)	
**Weight**	76 [67–84.5] (51–115)	79 [63.5–89.8] (54–122)	0.6254	75.5 [65.5–88.5] (51–112)	77 [62.5–88] (54–122)	0.8323	76.8 [68–86] (51–115)	75 [64.5–92.5] (54–122)	0.9617	76.5 [67–87] (51–115)	75 [63–90] (54–122)	0.9706
**BMI (median)**	25.7 [23.5–28.7] (20.9–35.4)	27.4 [23.4–31] (20.5–43)	0.1863	27.1 [23.7–29.6] (21–35.4)	27.2 [23.1–30.8] (10.5–42.9)	0.7574	26 [24.2–29] (20.9–35.4)	27.4 [23.5–31.8] (21–42.9)	0.2055	27.2 [24–29.8] (21–35.4)	27.3 [23.3–31.7] (21–42.9)	0.7067
**Localization**			0.1601			0.1188			0.1799			0.1486
Rectum	34 (42.5%)	24 (55.8%)		15 (37.5%)	22 (55%)		33 (45.8%)	22 (59.5%)		14 (41.2%)	20 (58.8%)	
Colon	46 (57.5%)	19 (44.2%)		25 (62.5%)	18 (45%)		39 (54.2%)	15 (40.5%)		20 (58.8%)	14 (41.2%)	
**Type of procedure**			0.2756			0.3668			0.3101			0.2441
ASR	5 (6.2%)	6 (14%)		2 (5%)	6 (15%)		5 (6.9%)	5 (13.5%)		2 (5.9%)	5 (14.7%)	
Left hemicolectomy	2 (2.5%)	1 (2.3%)		1 (2.5%)	1 (2.5%)		2 (2.8%)	0 (0%)		1 (2.9%)	0 (0%)	
Right hemicolectomy	31 (38.7%)	16 (37.2%)		18 (45%)	15 (37.5%)		25 (34.7%)	13 (35.1%)		13 (38.2%)	12 (35.3%)	
Anterior rectal resection	36 (45%)	20 (46.5%)		17 (42.5%)	18 (45%)		35 (48.6%)	19 (51.4%)		15 (44.1%)	17 (50%)	
Sigmoidectomy	6 (7.5%)	0 (0%)		2 (5%)	0 (0%)		5 (6.9%)	0 (0%)		3 (8.8%)	0 (0%)	
**Procedure**			0.2291			0.3105			0.3566			0.4742
Stoma	6 (7.5%)	7 (16.3%)		3 (7.5%)	7 (17.5%)		6 (8.3%)	6 (16.2%)		3 (8.8%)	6 (17.6%)	
Anastomosis	74 (92.5%)	36 (83.7%)		37 (92.5%)	33 (82.5%)		66 (91.7%)	31 (83.8%)		31 (91.2%)	28 (82.4%)	
**Anastomosis**			<0.0001 *			<0.0001 *			<0.0001 *			0.0001 *
Mechanical	37 (50%)	33 (97.1%)		18 (48.6%)	31 (96.9%)		35 (53%)	28 (96.6%)		15 (48.4%)	26 (96.3%)	
Hend-sewn	37 (50%)	1 (2.9%)		19 (51.4%)	1 (3.1%)		31 (47%)	1 (3.4%)		16 (51.6%)	1 (3.7%)	
**Type of anastomosis**			<0.0001 *			<0.0001 *			<0.0001 *			<0.0001 *
Extracorporeal	66 (89.2%)	1 (2.8%)		30 (81.1%)	1 (3%)		59 (89.4%)	1 (3.2%)		27 (87.1%)	1 (3.6%)	
Intracorporeal	8 (10.8%)	35 (97.2%)		7 (18.9%)	32 (97%)		7 (10.6%)	30 (96.8%)		4 (12.9%)	27 (96.4%)	
**Protective ileostomy**			0.0624			0.1653			0.0577			0.1735
No	74 (100%)	41 (95.3%)		38 (100%)	38 (95%)		66 (100%)	35 (94.6%)		31 (100%)	32 (94.1%)	
Yes	0 (0%)	2 (4.7%)		0 (0%)	2 (5%)		0 (0%)	2 (5.4%)		0 (0%)	2 (5.9%)	
**Leak test**			0.4720			0.3586			0.4055			0.4653
No	50 (62.5%)	24 (55.8%)		27 (67.5%)	23 (57.5%)		43 (59.7%)	19 (51.4%)		21 (61.8%)	18 (52.9%)	
Yes	30 (37.5%)	19 (44.2%)		13 (32.5%)	17 (42.5%)		29 (40.3%)	18 (48.6%)		13 (38.2%)	16 (47.1%)	
**Intraoperative complications**			0.6542			0.0794			0.6300			0.3173
No	79 (98.7%)	42 (97.7%)		36 (90%)	30 (75%)		71 (98.6%)	36 (97.3%)		34 (100%)	33 (97.1%)	
Yes	1 (1.3%)	1 (2.3%)		4 (10%)	10 (25%)		1 (1.4%)	1 (2.7%)		0 (0%)	1 (2.9%)	
**Postoperative leak**			0.0890			0.3079			0.2270			0.1542
No	79 (98.7%)	40 (93%)		39 (97.5%)	37 (92.5%)		71 (98.6%)	35 (94.6%)		34 (100%)	32 (94.1%)	
Yes	1 (1.3%)	3 (7%)		1 (2.5%)	3 (7.5%)		1 (1.4%)	2 (5.4%)		0 (0%)	2 (5.9%)	
**Reintervention**			0.6723			0.3079			0.7016			1.0000
No	77 (96.2%)	42 (97.7%)		37 (92.5%)	39 (97.5%)		69 (95.8%)	36 (97.3%)		33 (97.1%)	33 (97.1%)	
Yes	3 (3.7%)	1 (2.3%)		3 (7.5%)	1 (2.5%)		3 (4.2%)	1 (2.7%)		1 (2.9%)	1 (2.9%)	
**ICU stay**			0.2455			0.5587			0.2270			0.1542
No	79 (98.7%)	41 (95.3%)		39 (97.5%)	38 (95%)		71 (98.6%)	35 (94.6%)		34 (100%)	32 (94.1%)	
Yes	1 (1.3%)	2 (4.7%)		1 (2.5%)	2 (5%)		1 (1.4%)	2 (5.4%)		0 (0%)	2 (5.9%)	
**Postoperative complications**			0.0062 *			0.0794			0.0258 *			0.2066
No	75 (93.7%)	33 (76.7%)		36 (90%)	30 (75%)		67 (93.1%)	29 (78.4%)		30 (88.2%)	26 (76.5%)	
Yes	5 (6.2%)	10 (23.3%)		4 (10%)	10 (25%)		5 (6.9%)	8 (21.6%)		4 (11.8%)	8 (23.5%)	
**CCI**	8.7 [8.7–8.7] (8.7–66.9)	0 [0–0] (0–100)	0.0001 *	8.7 [8.7–8.7] (8.7–66.9)	0 [0–20.9] (0–100)	0.0003 *	8.7 [8.7–8.7] (8.7–66.9)	0 [0–20.9] (0–100)	0.0001 *	8.7 [8.7–8.7] (8.7–45.7)	0 [0–20.9] (0–100)	0.0001 *
**Blood transfusion**			0.1247			0.2422			0.1759			0.5516
No	70 (87.5%)	33 (76.7%)		35 (87.5%)	31 (77.5%)		62 (86.1%)	28 (75.7%)		28 (82.4%)	26 (76.5%)	
Yes	10 (12.5%)	10 (23.3%)		5 (12.5%)	9 (22.5%)		10 (13.9%)	9 (24.3%)		6 (17.6%)	8 (23.5%)	
**Length of stay**	5 [4–6] (2–22)	4.5 [4–6] (3–29)	0.1140	5 [5–6.5] (2–22)	4 [4–6] (3–29)	0.0827	5 [4.5–6] (2–22)	4 [4–6] (3–29)	0.0123 *	5 [5–6] (2–12)	4 [4–6] (3–29)	0.0188 *
**Grading**			0.0739			0.0705			0.0727			0.3111
G1	45 (63.4%)	25 (61%)		23 (67.6%)	24 (61.5%)		41 (62.1%)	21 (58.3%)		18 (60%)	20 (58.8%)	
G2	14 (19.7%)	3 (7.3%)		7 (20.6%)	3 (7.7%)		14 (21.2%)	3 (8.3%)		6 (20%)	3 (8.8%)	
G3	12 (16.9%)	13 (31.7%)		4 (11.8%)	12 (30.8%)		11 (16.7%)	12 (33.3%)		6 (20%)	11 (32.4%)	
**cT**			0.3360			0.1236			0.4015	3 (9.1%) 7 (21.2%) 16 (48.5%) 7 (21.2%)	1 (2.9%) 7 (20.6%) 22 (64.7%) 4 (11.8%)	0.4316
cTx	0 (0%)	1 (2.5%)		0 (0%)	1 (2.6%)		0 (0%)	0 (0%)				
cT1	6 (7.9%)	1 (2.5%)		3 (8.1%)	1 (2.6%)		6 (8.6%)	1 (2.8%)				
cT2	23 (30.3%)	11 (27.5%)		13 (35.1%)	10 (26.3%)		18 (25.7%)	8 (22.2%)				
cT3	35 (46.1%)	23 (57.5%)		12 (32.4%)	22 (57.9%)		34 (48.6%)	23 (63.9%)				
cT4	12 (15.8%)	4 (10%)		9 (24.3%)	4 (10.5%)		12 (17.1%)	4 (11.1%)				
**cN**			0.1573			0.3049			0.1573			0.3173
cN0	0 (0%)	1 (2.5%)		0 (0%)	1 (2.6%)		0 (0%)	1 (2.8%)		0 (0%)	1 (2.9%)	
cNx	80 (100%)	39 (97.5%)		40 (100%)	37 (97.4%)		72 (100%)	35 (97.2%)		34 (100%)	33 (97.1%)	
**TRG**			0.0718			0.9053			0.1142			0.2269
TRG1	2 (28.6%)	2 (5.7%)		0 (0%)	2 (6.1%)		2 (28.6%)	2 (6.9%)		1 (50%)	2 (7.4%)	
TRG2	1 (14.3%)	15 (42.9%)		1 (33.3%)	14 (42.4%)		1 (14.3%)	12 (41.4%)		1 (50%)	11 (40.7%)	
TRG3	1 (14.3%)	12 (34.3%)		1 (33.3%)	11 (33.3%)		1 (14.3%)	10 (34.5%)		0 (0%)	9 (33.3%)	
TRG4	3 (42.9%)	6 (17.1%)		1 (33.3%)	6 (18.2%)		3 (42.9%)	5 (17.2%)		0 (0%)	5 (18.5%)	
**pT**			0.3016			0.5908			0.3704			0.5908
pT1	0 (0%)	1 (2.3%)		0 (0%)	1 (2.5%)		0 (0%)	1 (2.7%)		0 (0%)	1 (2.9%)	
pT2	24 (30%)	10 (23.3%)		10 (25%)	9 (22.5%)		21 (29.2%)	10 (27%)		10 (29.4%)	9 (26.5%)	
pT3	56 (70%)	32 (74.4%)		30 (75%)	30 (75%)		51 (70.8%)	26 (70.3%)		24 (70.6%)	24 (70.6%)	
**pN**			0.1726			0.9195			0.4590			0.9195
pN0	80 (100%)	42 (97.7%)		28 (70%)	28 (70%)		42 (58.2%)	23 (62.2%)		22 (64.7%)	22 (64.7%)	
pN1	0 (0%)	0 (0%)		7 (17.5%)	6 (15%)		19 (26.4%)	7 (18.9%)		7 (20.6%)	6 (17.6%)	
pN2	0 (0%)	0 (0%)		5 (12.5%)	6 (15%)		11 (15.3%)	6 (16.2%)		5 (14.7%)	6 (17.6%)	
pNx	0 (0%)	1 (2.3%)		0 (0%)	0 (0%)		0 (0%)	1 (2.7%)		0 (0%)	0 (0%)	
**Number of collected lymph nodes**	9.5 [5–14] (1–39)	9.5 [4–13] (0–28)	0.8472	11 [6.5–14.5] (1–39)	10 [4.5–13] (0–28)	0.4471	9 [5–14] (1–39)	10 [4–13.3] (0–28)	0.8331	9 [5–14.3] (1–39)	10 [4–13] (0–28)	0.8358
**Number of metastatic lymph nodes**	0 [0–2] (0–29)	0 [0–2] (0–13)	0.6774	0 [0–0.5] (0–29)	0 [0–2.8] (0–13)	0.3188	0 [0–2] (0–29)	0 [0–2.3] (0–13)	0.7862	0 [0–1.3] (0–29)	0 [0–3] (0–13)	0.4172
**Surgical margins**			0.3441			0.3173			0.3441			0.3173
R0	68 (94.4%)	37 (100%)		33 (97.1%)	34 (100%)		68 (94.4%)	37 (100%)		33 (97.1%)	34 (100%)	
R1	3 (4.2%)	0 (0%)		1 (2.9%)	0 (0%)		3 (4.2%)	0 (0%)		1 (2.9%)	0 (0%)	
R2	1 (1.4%)	0 (0%)		0 (0%)	0 (0%)		1 (1.4%)	0 (0%)		0 (0%)	0 (0%)	
**Procedure time (min)**	170 [142.5–207.5] (95–275)	160 [140.5–213.8] (55–290)	0.8443	180 [150–210] (120–270)	158 [142–205] (55–290)	0.2936	175 [145–210] (95–275)	160 [136.8–215] (55–290)	0.6286	177.5 [150–220] (115–270)	158 [139–210] (55–290)	0.3140
**Dead/alive**			0.1726			0.3173			0.1630			0.3173
Dead	0 (0%)	1 (2.3%)		0 (0%)	1 (2.5%)		0 (0%)	1 (2.7%)		0 (0%)	1 (2.9%)	
Alive	80 (100%)	42 (97.7%)		40 (100%)	39 (97.5%)		72 (100%)	36 (97.3%)		34 (100%)	33 (97.1%)	
**TO**			0.0232 *			0.1745			0.1281			0.1259
No	8 (10%)	11 (25.6%)		6 (15%)	11 (27.5%)		8 (11.1%)	9 (24.3%)		4 (11.8%)	9 (26.5%)	
Yes	72 (90%)	32 (74.4%)		34 (85%)	29 (72.5%)		64 (88.9%)	28 (75.7%)		30 (88.2%)	25 (73.5%)	
**TOO**			0.1328			0.0396 *			0.1328			0.0396 *
No	19 (26.4%)	15 (44.1%)		7 (20.6%)	15 (44.1%)		19 (26.4%)	15 (44.1%)		7 (20.6%)	15 (44.1%)	
Yes	53 (70.7%)	22 (29.3%)		27 (79.4%)	19 (55.9%)		53 (70.7%)	22 (29.3%)		27 (79.4%)	19 (55.9%)	

*—statistically significant result. Abbreviations: ASR—abdominosacral resection, BMI—Body Mass Index, CCI—comprehensive complication index, ICU—intensive care unit, IQR—interquartile range, LOS—length of stay, TO—textbook outcome, TOO—textbook oncological outcome, TRG—tumour regression grade.

**Table 2:** The impact of demographic and clinical factors on the likelihood of achieving TO or TOO. Laparoscopic group (after PSM).

Variable	TO [n = 40] N (%) or Median [IQR] (Min.–Max.)		Univariable	Multivariable	TOO [n = 34] N (%) or Median [IQR] (Min.–Max.)		Univariable	Multivariable
			OR [95%CI] *p*	OR [95%CI] *p*			OR [95%CI] *p*	OR [95%CI] *p*
	No [N = 6; 15%]	Yes [N = 34; 85%]			No [N = 7; 20.6%]	Yes [N = 27; 79.4%]		
**Sex**			0.14 [0.01–1.33] 0.0872	0.15 [0.01–1.94] 0.1449			0.71 [0.32–1.57] 0.3991	0.46 [0.05–4.14] 0.4858
Female	1 (4.8%)	20 (95.2%)			3 (15.8%)	16 (84.2%)		
Male	5 (26.3%)	14 (73.7%)			4 (26.7%)	11 (73.3%)		
**Age**			7.14 [0.75–67.98] 0.0872	3.43 [0.23–50.71] 0.3694			2.33 [0.70–7.76] 0.1668	4.21 [0.45–39.06] 0.2063
<65 years	5 (26.3%)	14 (73.7%)			5 (35.7%)	9 (64.3%)		
≥65 years	1 (4.8%)	20 (95.2%)			2 (10%)	18 (90%)		
**BMI**			0.94 [0.17–5.36] 0.9456	0.69 [0.05–9.58] 0.9973			0.24 [0.04–1.45] 0.1184	0.96 [0.09–10.29] 0.9763
Correct weight	3 (15%)	17 (85%)			2 (10.5%)	17 (89.5%)		
Overweight or obese	3 (15.8%)	16 (84.2%)			5 (33.3%)	10 (66.7%)		
**Location**			1.24 [0.20–7.74] 0.8194	1.73 [0.11–27.68] 0.6970			8.59 [0.57–128.70] 0.1193	N/d 0.9982
Rectum	2 (13.3%)	13 (86.7%)			0 (0%)	14 (100%)		
Colon	4 (16%)	21 (84%)			7 (36.8%)	12 (63.2%)		
**Anastomosis/stoma**			1.44 [0.07–31.47] 0.8151	N/d 0.9992			0.64 [0.34–1.22] 0.1737	N/d 0.9999
Stoma	0 (0%)	3 (100%)			0 (0%)	3 (100%)		
Anastomosis	6 (16.2%)	31 (83.8%)			7 (22.6%)	24 (77.4%)		
**Anastomosis**			0.47 [0.07–2.94] 0.4190	1.58 [0.05–46.13] 0.9984			0.34 [0.05–2.10] 0.2449	1.31 [0.12–14.39] 0.8242
Mechanical	2 (11.1%)	16 (88.9%)			2 (13.3%)	13 (86.7%)		
Hand-sewn	4 (21.1%)	15 (78.9%)			5 (31.2%)	11 (68.7%)		
**Type of anastomosis**			0.83 [0.08–8.52] 0.8778	1.58 [0.07–0.79] 0.7903			1.28 [0.79–2.08] 0.3122	1.31 [0.12–14.39] 0.8242
Extracorporeal	5 (16.7%)	25 (83.3%)			5 (18.5%)	22 (81.5%)		
Intracorporeal	1 (14.3%)	6 (85.7%)			2 (50%)	2 (50%)		
**Leak test**			2.73 [0.28–26.12] 0.3841	2.40 [0.11–52.36] 0.5768			7.71 [0.51–116.07] 0.0805	N/d 0.9983
No	5 (18.5%)	22 (81.5%)			7 (33.3%)	14 (66.7%)		
Yes	1 (7.7%)	12 (92.3%)			0 (0%)	13 (100%)		
**CCI (median)**	37.7 [27.6–45.7] (8.7–66.9)	8.7 [8.7–8.7] (8.7–22.6)	0.0001 *	N/a	27.6 [12.2–33] (8.7–45.7%)	8.7 [8.7–8.7] (8.7–22.6)	0.0003 *	N/a
**Blood transfusion**			0.19 [0.02–1.53] 0.1200	N/d 0.9985			0.52 [0.12–2.28] 0.3844	0.42 [0.01–18.89] 0.6560
No	4 (11.4%)	31 (88.6%)			5 (17.9%)	23 (82.1%)		
Yes	2 (40%)	3 (60%)			2 (33.3%)	4 (66.7%)		
**Length of stay**			18.82 [0.68–523.01] 0.0836	N/d 0.9992			N/d	N/d
≥15 days	1 (100%)	0 (0%)			7 (20.6%)	27 (79.4%)		
<15 days	5 (12.8%)	34 (87.2%)			0 (0%)	0 (0%)		
**Grading**			0.95 [0.15–6.17] 0.9549	4.15 [0.17–102.45] 0.3848			1.52 [0.43–5.37] 0.5139	5.23 [0.34–80.49] 0.2360
G1	4 (17.4%)	19 (82.6%)			5 (27.8%)	13 (72.2%)		
G2. G3	2 (18.2%)	9 (81.8%)			2 (16.7%)	10 (83.3%)		
**cT**			0.07 [0.01–1.39] 0.0818	N/d 0.9974			0.97 [0.57–1.65] 0.9087	1.28 [0.11–15.37] 0.8442
cT1. cT2	0 (0%)	16 (100%)			2 (20%)	8 (80%)		
cT3. cT4	6 (28.6%)	15 (71.4%)			5 (21.7%)	18 (78.3%)		
**pT**			1.63 [0.25–10.58] 0.6115	1.95 [0.10–39.36] 0.6617			1.30 [0.66–2.56] 0.4540	3.53 [0.23–53.42] 0.3636
pT1. pT2	2 (20%)	8 (80%)			3 (30%)	7 (70%)		
pT3	4 (13.3%)	26 (86.7%)			4 (16.7%)	20 (83.3%)		
**pN**			0.36 [0.06–2.12] 0.2586	0.86 [0.10–7.82] 0.8959			1.30 [0.36–4.62] 0.6889	2.92 [0.18–48.12] 0.4529
pN0	3 (10.7%)	25 (89.3%)			5 (22.7%)	17 (77.3%)		
pN1. pN2	3 (25%)	9 (75%)			2 (16.7%)	10 (83.3%)		
**Number of collected lymph nodes**	13.5 [10–15] (5–39)	10.5 [6–14] (1–30)	0.0688	N/a	14.5 [13–2} (5–39)	9 [6–11] (1–20)	0.0107 *	N/a
**Number of metastatic lymph nodes**	0 [0–2] (0–29)	0 [0–0] (0–14)	0.5578	N/a	0 [0–10.5] (0–29)	0 [0–1] (0–9)	0.6333	N/a
**Procedure time (min)**	205 [150–240] (150–240)	177.5 [150–210] (120–270)	0.2870	N/a	200 [150–240] (140–250)	180 [155–215] (120–270)	0.6624	N/a

*—statistically significant result. Abbreviations: CI—confidence interval, BMI—Body Mass Index, CCI—comprehensive complication index, IQR—interquartile range, N/a—not applicable, N/d—not determined, OR—odds ratio, TO—textbook outcome, TOO—textbook oncological outcome, TRG—tumour regression grade.

**Table 3:** The impact of demographic and clinical factors on the likelihood of achieving TO or TOO—robotic group (after PSM).

Variable	TO [n = 40] N (%) or Median [IQR] (Min.–Max.)		Univariable	Multivariable	TOO [n = 34] N (%) or Median [IQR] (Min.–Max.)		Univariable	Multivariable
			OR [95%CI] *p*	OR [95%CI] *p*			OR [95%CI] *p*	OR [95%CI] *p*
	No [N = 11; 27.5%]	Yes [N = 29; 72.5%]			No [N = 15; 44.1%]	Yes [N = 19; 55.9%]		
**Sex**			0.82 [0.42–1.61] 0.5682	0.62 [0.14–2.82] 0.5336			0.53 [0.24–1.15] 0.1069	0.16 [0.02–1.37] 0.0932
Female	5 (23.8%)	16 (76.2%)			6 (31.6%)	13 (68.4%)		
Male	6 (31.6%)	13 (68.4%)			9 (60%)	6 (40%)		
**Age**			0.57 [0.26–1.22] 0.1486	0.44 [0.10–2.02] 0.2926			0.68 [0.29–1.59] 0.3706	0.45 [0.06–3.14] 0.4198
<65 years	5 (20%)	20 (80%)			8 (38.1%)	13 (61.9%)		
≥65 years	6 (40%)	9 (60%)			7 (53.8%)	6 (46.2%)		
**BMI**			1.40 [0.30–6.49] 0.6645	1.25 [0.26–5.92] 0.7819			2.91 [0.61–13.83] 0.1794	1.25 [0.17–9.39] 0.8278
Correct weight	8 (29,6%)	19 (70,4%)			3 (27.3%)	8 (72.7%)		
Overweight or obese	3 (23,1%)	10 (76,9%)			12 (52.2%)	11 (47.8%)		
**Localisation**			0.81 [46–1.44] 0.4749	0.51 [0.08–3.18] 0.4735			1.37 [1.12–5.03] 0.0247 *	3.58 [0.42–30.72] 0.2445
Rectum	7 (31.8%)	15 (68.2%)			5 (25%)	15 (75%)		
Colon	4 (22.2%)	14 (77.8%)			10 (71.4%)	4 (28.6%)		
**Anastomosis/stoma**			0.51 [0.13–1.91] 0.3137	0.28 [0.01–14.47] 0.5308			1.58 [0.33–7.49] 0.5652	6.52 [0.05–940.15] 0.4599
Stoma	3 (42.9%)	4 (57.1%)			2 (33.3%)	4 (66.7%)		
Anastomosis	8 (24.2%)	25 (75.8%)			13 (46.4%)	15 (53.6%)		
**Anastomosis**			1.08 [0.05–24.18] 0.9613	N/d 0.9978			2.80 [0.12–63.20] 0.5173	N/d 0.9991
Mechanical	8 (25.8%)	23 (74.2%)			13 (50%)	13 (50%)		
Hand-sewn	0 (0%)	1 (100%)			0 (0%)	1 (100%)		
**Type of anastomosis**			1.04 [0.05–23.27] 0.9810	N/d 0.9979			2.63 [0.12–59.40] 0.5442	N/d 0.9991
Extracorporeal	0 (0%)	1 (100%)			0 (0%)	1 (100%)		
Intracorporeal	8 (25%)	24 (75%)			13 (48.1%)	14 (51.9%)		
**Protective ileostomy**			0.38 [0.03–5.55] 0.4789	0.69 [0.03–15] 0.8125			0.79 [0.05–11.61] 0.8631	0.62 [0.03–14.34] 0.7636
No	10 (26.3%)	28 (73.7%)			14 (43.7%)	18 (56.2%)		
Yes	1 (50%)	1 (50%)			1 (50%)	1 (50%)		
**Leak test**			0.91 [0.42–1.98] 0.8132	1.17 [0.22–6.35] 0.8560			2.37 [0.96–5.87] 0.0624	2.94 [0.34–25.42] 0.3267
No	6 (26.1%)	17 (73.9%)			11 (61.1%)	7 (38.9%)		
Yes	5 (29.4%)	12 (70.6%)			4 (25%)	12 (75%)		
**CCI (median)**	20.9 [20.9–75.5] (0–100)	0 [0–0] (0–20.9)	<0.0001 *	N/a	20.9 [0–20.9] (0–100)	0 [0–0] (0–20.9)	0.0017 *	N/a
**Blood transfusion**			0.05 [0.01–0.34] 0.0023	N/d 0.9978			0.11 [0.02–0.82] 0.0310	0.05 [0.01–1.10] 0.9980
No	3 (9.7%)	28 (90.3%)			8 (30.8%)	18 (69.2%)		
Yes	8 (88.9%)	1 (11.1%)			7 (87.5%)	1 (12.5%)		
**Length of stay**			1.11 [0.90–1.37] 0.3175	N/d 0.9980			1.08 [0.93–1.25] 0.3174	N/d 0.9992
<15 days	1 (100%)	0 (0%)			1 (100%)	0 (0%)		
≥15 days	9 (23.7%)	29 (76.3%)			13 (40.6%)	19 (59.4%)		
**Grading**			1.57 [0.55–4.52] 0.4013	3.91 [0.59–25.69] 0.1562			1.97 [0.77–5.06] 0.1569	4.59 [0.58–36.31] 0.1488
G1	8 (33.3%)	16 (66.7%)			11 (55%)	9 (45%)		
G2. G3	3 (20%)	12 (80%)			4 (28.6%)	10 (71.4%)		
**cT**			0.80 [0.54–1.19] 0.2656	0.39 [0.06–2.61] 0.3304			0.58 [0.39–0.85] 0.0052 *	N/d 0.9982
cT1. cT2	2 (18.2%)	9 (81.8%)			0 (0%)	8 (100%)		
cT3. cT4	9 (34.6%)	17 (65.4%)			15 (57.7%)	11 (42.3%)		
**TRG**			0.90 [0.44–1.83] 0.7706	0.82 [0.11–6.02] 0.8444			0.80 [0.39–1.65] 0.5447	0.38 [0.03–5.39] 0.4782
TRG1. TRG2	4 (25%)	12 (75%)			5 (38.5%)	8 (61.5%)		
TRG3. TRG4	5 (29.4%)	12 (70.6%)			7 (50%)	7 (50%)		
**pT**			0.76 [0.56–1.03] 0.0782	0.04 [0.01–1.58] 0.0869			1.11 [0.71–1.73] 0.6611	0.21 [0.12–3.72] 0.2880
pT1. pT2	1 (10%)	9 (90%)			5 (50%)	5 (50%)		
pT3	10 (33.3%)	20 (66.7%)			10 (41.7%)	14 (58.3%)		
**pN**			0.76 [0.28–2.02] 0.5803	1.08 [0.22–5.35] 0.9245			1.12 [0.44–2.79] 0.8324	1.53 [0.25–9.37] 0.6445
pN0	7 (25%)	21 (75%)			10 (45.5%)	12 (54.5%)		
pN1. pN2	4 (33.3%)	8 (66.7%)			5 (41.7%)	7 (58.3%)		
**Number of collected lymph nodes**	9 [2.3–12] (0–25)	10 [6–15] (0–28)	0.3274	N/a	12 [6.8–22.8] (0–28)	9 [4–11.8] (0–16)	0.0009 *	N/a
**Number of metastatic lymph nodes**	0 [0–6] (0–9)	0 [0–2] (0–13)	0.4592	N/a	0 [0–2.8] (0–9)	0 [0–2.8] (0–13)	0.6770	N/a
**Procedure time (min)**	195 [147.5–243.8] (115–255)	155 [127.5–196.3] (55–290)	0.0922	N/a	185 [146.3–222.5] (115–255)	155 [122.5–187.5] (55–290)	0.1264	N/a

*—statistically significant result. Abbreviations: CI—confidence interval, BMI—Body Mass Index, CCI—comprehensive complication index, IQR—interquartile range, N/a—not applicable, N/d—not determined, OR—odds ratio, TO—textbook outcome, TOO—textbook oncological outcome, TRG—tumour regression grade.

**Table 4:** The impact of composite measure components on the likelihood of achieving TO or TOO—laparoscopic group (after PSM).

Variable	TO [n = 40] N (%)		OR [95%CI] *p*	TOO [n = 34] N (%)		OR [95%CI] *p*
	No [N = 6; 15%]	Yes [N = 34; 85%]		No [N = 7; 20.6%]	Yes [N = 27; 79.4%]	
**1. Positive margins**			0.08 [0.01–1.77] 0.1102			0.15 [0.01–3.33] 0.2280
No	5 (15.2%)	28 (84.8%)		6 (18.2%)	27 (81.8%)	
Yes	1 (100%)	0 (0%)		1 (100%)	0 (0%)	
**2. ICU stay**			0.07 [0.01–1.47] 0.0865			0.15 [0.01–3.33] 0.2280
No	5 (12.8%)	34 (87.2%)		7 (20.6%)	27 (79.4%)	
Yes	1 (100%)	0 (0%)		0 (0%)	0 (0%)	
**3. Intraoperative complications**			0.07 [0.01–1.47] 0.0865			0.15 [0.01–3.33] 0.2280
No	5 (12.8%)	34 (87.2%)		7 (20.6%)	27 (79.4%)	
Yes	1 (100%)	0 (0%)		0 (0%)	0 (0%)	
**4. Postoperative complications**			0.02 [0.01–0.42] 0.0103 *			0.06 [0.01–1.01] 0.0507
No	2 (5.6%)	34 (94.4%)		3 (10%)	27 (90%)	
Yes	3 (100%)	0 (0%)		4 (100%)	0 (0%)	
**5. Length of stay ≥ 15 days**			1.20 [0.84–1.72] 0.3180			1.11 [0.90–1.37] 0.3175
No	5 (12.8%)	34 (87.2%)		7 (20.6%)	27 (79.4%)	
Yes	1 (100%)	0 (0%)		0 (0%)	0 (0%)	
**6. Reintervention**			0.03 [0.01–0.49] 0.0145 *			0.06 [0.01–1.12] 0.0594
No	3 (8.1%)	34 (91.9%)		6 (18.2%)	27 (81.8%)	
Yes	3 (100%)	0 (0%)		1 (100%)	0 (0%)	
**7. Retrieval of the appropriate number of lymph nodes ^#^**			1.68 [0.74–3.79] 0.2135			3.33 [1.29–8.59] 0.0127 *
No	3 (37.5%)	5 (62.5%)		6 (100%)	0 (0%)	
Yes	3 (10.3%)	26 (89.7%)		1 (3.6%)	27 (96.4%)	
**8. Alive**			5.31 [0.10–292.29] 0.4144			3.67 [0.07–200.65] 0.5246
No	0 (0%)	0 (0%)		0 (0%)	0 (0%)	
Yes	6 (15%)	34 (85%)		7 (20.6%)	27 (79.4%)	

^#^—for colon ≥ 12, irrelevant for colon, *—statistically significant result. Abbreviations: CI—confidence interval, ICU—Intensive care unit, OR—odds ratio, TO—textbook outcome, TOO—textbook oncological outcome.

**Table 5:** The impact of composite measure components on the likelihood of achieving TO or TOO—robotic group (after PSM).

Variable	TO [n = 40] N (%)		OR [95%CI] *p*	TOO [n = 34] N (%)		OR [95%CI] *p*
	No [N = 11; 27.5%]	Yes [N = 29; 72.5%]		No [N = 15; 44.1%]	Yes [N = 19; 55.9%]	
**1. Positive margins**			0.37 [0.01–20.14] 0.6277			0.79 [0.01–42.38] 0.9099
No	9 (26.5%)	25 (73.5%)		15 (44.1%)	19 (55.9%)	
Yes	0 (0%)	0 (0%)		0 (0%)	0 (0%)	
**2. ICU stay**			0.08 [0.01–1.55] 0.0946			0.16 [0.01–3.10] 0.2256
No	9 (23.7%)	29 (76.3%)		13 (40.6%)	19 (59.4%)	
Yes	2 (100%)	0 (0%)		2 (100%)	0 (0%)	
**3. Intraoperative complications**			0.36 [0.01–19.10] 0.6112			0.74 [0.01–39.73] 0.8840
No	10 (25.6%)	29 (74.4%)		14 (42.4%)	19 (67.6%)	
Yes	0 (0%)	0 (0%)		0 (0%)	0 (0%)	
**4. Postoperative complications**			0.02 [0.01–0.30] 0.0049 *			0.05 [0.01–0.76] 0.0309 *
No	1 (3.3%)	29 (96.7%)		7 (26.9%)	19 (73.1%)	
Yes	10 (100%)	0 (0%)		8 (100%)	0 (0%)	
**5. Length of stay ≥ 15 days**			0.11 [0.90–1.37] 0.3175			1.08 [0.93–1.25] 0.3174
No	9 (23.7%)	29 (76.3%)		13 (40.6%)	19 (59.4%)	
Yes	1 (100%)	0 (0%)		1 (100%)	0 (0%)	
**6. Reintervention**			0.13 [0.01–3.05] 0.2070			0.27 [0.01–6.12] 0.4082
No	10 (25.6%)	29 (74.4%)		14 (42.4%)	19 (57.6%)	
Yes	1 (100%)	0 (0%)		1 (100%)	0 (0%)	
**7. Retrieval of the appropriate number of lymph nodes ^#^**			0.85 [0.64–1.12] 0.2448			1.88 [1.17–3.01] 0.0092 *
No	1 (14.3%)	6 (85.7%)		7 (100%)	0 (0%)	
Yes	10 (33.3%)	20 (66.7%)		8 (29.6%)	19 (70.4%)	
**8. Alive**			1.10 [0.92–1.33] 0.3175			1.07 [0.94–1.23] 0.3174
No	1 (100%)	0 (0%)		1 (100%)	0 (0%)	
Yes	10 (25.6%)	29 (74.4%)		14 (42.4%)	19 (57.6%)	

^#^—for colon ≥ 12, irrelevant for colon, *—statistically significant result. Abbreviations: CI—confidence interval, ICU—Intensive care unit, OR—odds ratio, TO—textbook outcome, TOO—textbook oncological outcome.

## Data Availability

The data presented in this study are available on request from the corresponding author (accurately indicate status).

## Abbreviations

ASR	abdominosacral resection
BMI	body mass index
CI	confidence interval
CC	colon cancer
CCI	comprehensive complication index
CRC	colorectal cancer
CME	complete mesocolic excision
ERAS	enhanced recovery after surgery
HDI	human development index
ICU	intensive care unit
IQR	interquartile range
LOS	length of stay
MDT	multidisciplinary team
OS	overall survival
OR	odds ratio
PSM	propensity score matching
RAS	robot-assisted surgery
RC	rectal cancer
RCT	randomized controlled trial
STROBE	strengthening the reporting of observational studies in epidemiology
TME	total mesorectal excision
TNT	total neoadjuvant treatment
TO	textbook outcome
TOO	textbook oncological outcome
TRG	tumour regression grade
